# Assessing the effects of fluids and antibiotics in an acute murine model of sepsis: study protocol for the National Preclinical Sepsis Platform-01 (NPSP-01) Study

**DOI:** 10.12688/f1000research.163800.1

**Published:** 2025-05-01

**Authors:** Forough Jahandideh, Asher A. Mendelson, Patricia C. Liaw, Sean E. Gill, Stephane Bourque, Alison E. Fox-Robichaud, Gediminas Cepinskas, Kimberly F. Macala, Janet Sunohara- Neilson, Doreen Engelberts, David Sontag, Dhruva J. Dwivedi, Ian-Ling Yu, Onon Batnyam, Kashimbi Mbuta, Dean A. Fergusson, Monica Taljaard, Braedon McDonald, Manoj M. Lalu

**Affiliations:** 1 Blueprint Translational Research Group, Acute Care Research and Methods and Implementation Research Programs, Ottawa Hospital Research Institute, Ottawa, ON, Canada; 2 Department of Internal Medicine, Section of Critical Care, University of Manitoba, Winnipeg, Manitoba, Canada; 3 Department of Medicine, McMaster University, Hamilton, Ontario, Canada; 4 Department of Physiology and Pharmacology, Western University Schulich School of Medicine & Dentistry, London, Ontario, Canada; 5 Division of Respirology, Western University Schulich School of Medicine & Dentistry, London, Ontario, Canada; 6 Centre for Critical Illness Research, London Health Sciences Centre Research Institute, London, ON, Canada; 7 Department of Anesthesiology and Pain Medicine, University of Alberta, Edmonton, Alberta, Canada; 8 Department of Anatomy & Cell Biology, Western University Schulich School of Medicine & Dentistry, London, Ontario, Canada; 9 Department of Medical Biophysics, Western University Schulich School of Medicine & Dentistry, London, Ontario, Canada; 10 Department of Critical Care Medicine, Royal Alexandra Hospital, University of Alberta, Edmonton, AB, Canada; 11 Animal care services, Office of Research, University of Guelph, Guelph, ON, Canada; 12 Department of Critical Care Medicine, University of Calgary Cumming School of Medicine, Calgary, Alberta, Canada; 13 Methods and Implementation Research Program, Ottawa Hospital Research Institute, Ottawa, Ontario, Canada; 14 Faculty of Medicine, University of Ottawa, Ottawa, Ontario, Canada; 15 Department of Cellular and Molecular Medicine, University of Ottawa, Ottawa, Ontario, Canada; 16 Department of Anesthesiology and Pain Medicine, The Ottawa Hospital, University of Ottawa, Ottawa, Ontario, Canada

**Keywords:** Acute Sepsis, Antibiotic therapy, Feasibility, Fluid resuscitation, Inter-laboratory variability, Multicenter study, National Preclinical Sepsis Platform

## Abstract

**Background:**

Sepsis remains a leading cause of mortality in critical care. Despite extensive preclinical research on sepsis pathophysiology, the development of effective therapies has been largely unsuccessful. Key obstacles include limited construct validity of animal models, insufficient methodological rigor and the lack of collaborative frameworks akin to clinical trials. These issues plague not only sepsis research, but preclinical research in general. The National Preclinical Sepsis Platform (NPSP), an interdisciplinary network under Sepsis Canada, addresses these challenges in sepsis research through multilaboratory, randomized, controlled preclinical studies. NPSP-01 will establish baseline conditions for future investigations using an acute fecal-induced peritonitis model of sepsis.

**Methods:**

This randomized, controlled study will evaluate the effect of standard sepsis therapy in a mouse model of sepsis across six centres. Interlaboratory variability and the interaction of biological sex on outcomes will also be examined. C57BL/6 mice of both sexes will be randomized into sham (healthy control) + treatment, sepsis, or sepsis + treatment groups. Sepsis will be induced via intraperitoneal injection of fecal slurry, while sham mice will receive vehicle control. Antibiotics and fluids will be administered to treatment groups at 4 hours post-induction, and mice with be euthanized at 8 hours post-induction. The primary outcome is plasma interleukin-6 levels. Secondary outcomes include biological (blood gas and chemistry, white blood cell count, bacterial load), clinical (body weight, core temperature, sepsis score, mortality as measured by surrogate humane endpoints), and feasibility measures.

**Conclusions:**

NPSP-01 will be the first multilaboratory study of sepsis and represents a shift in preclinical critical illness research, mirroring the rigor of clinical multicenter trials. By addressing procedural standardization, interlaboratory variability, and sex-based differences, this study aims to enhance the reliability and translational relevance of preclinical findings. The outcomes of NPSP-01 will establish foundational data for future investigations and provide a roadmap for rigorous collaborative preclinical studies to accelerate the evaluation of novel sepsis therapies.

**Registration:**

PreclinicalTrials.eu PCTE0000552

Protocol Version 1.0, October 21, 2024

## Introduction

Preclinical research has significantly advanced our understanding of sepsis pathophysiology, shedding light on key mediators such as inflammatory cytokine production (e.g., interleukin [IL]6, tumor necrosis factor [TNF]α) and mechanisms of organ dysfunction (e.g., mitochondrial failure, endothelial permeability, immunothrombosis). Despite these insights, septic shock mortality rates persist at 25 – 30%, ^1^ and decades of research have failed to translate findings from preclinical models into effective therapies. This translational challenge is not unique to sepsis: across biomedical fields, fewer than 5% of high-impact preclinical findings are translated from “bench-to-bedside”. ^2, 3^

Several modifiable factors could improve translational success in sepsis research. First, construct/translational validity ^1^ of animal models can be augmented to mimic some of the complexity and heterogeneity of human sepsis. For instance, researchers could incorporate standard clinical interventions, such as fluid resuscitation and antibiotics, use true bacterial or viral pathogens instead of surrogates like lipopolysaccharide, and study both male and female animals within a wider range of age and genetic diversity. ^2– 4^ In addition, methodological rigour could be improved to reduce risk of bias (e.g., through best practices like randomization and blinding) and ensure adequate statistical power (e.g., through sample size calculations). Other factors such as variability in laboratory conditions and protocols, lack of transparency (e.g., insufficient methods details provided), and different baseline conditions, contribute to variability in findings between laboratories. ^5^ Addressing these challenges requires a coordinated and collaborative approach.

A promising strategy to increase the translational potential of preclinical findings is the use of multilaboratory collaborative studies. While multicenter studies are a gold standard approach in clinical research, they remain underutilized in preclinical settings. ^6^ To address this gap in sepsis research, we established the National Preclinical Sepsis Platform (NPSP), an interdisciplinary network of scientists, veterinarians, and interest holders under Sepsis Canada. ^7^ Our objective is to conduct randomized, controlled, multilaboratory studies in sepsis, employing harmonized protocols across several laboratories. This will establish a network capable of conducting rigorous studies that can accelerate testing of promising therapies for sepsis.

For our initial study, NPSP-01, we are implementing procedural standardization and rigorous practices to establish baseline conditions for future investigations. Specifically, we will compare outcomes in six independent laboratories between untreated septic mice and those treated with clinical standard of care antibiotics and fluid resuscitation. We hypothesize that administering antibiotics and fluids will reduce systemic inflammation as assessed by plasma IL6, a clinically relevant pro-inflammatory cytokine and biomarker for sepsis. ^8^ We will also assess feasibility, interlaboratory variability, and the interaction of biological sex on study outcomes. To our knowledge, this will be the first multilaboratory preclinical study of critical illness in the world.

### Objectives

1. To use a multilaboratory approach to compare outcomes between untreated septic mice and septic mice treated with usual therapy (i.e., antibiotic and fluids).
2. To evaluate inter-laboratory variability in estimated treatment effects.
3. To evaluate biological sex as a potential effect modifier on study outcomes. ^7^
4. To assess the feasibility of conducting a multicenter preclinical sepsis study across six laboratories in Canada.

## Methods and design

Animal experiments will be conducted in accordance with the Canadian Council on Animal Care *Guide to Care and Use of Experimental Animals.* We have received approval from the institutional animal care and use committees at the University of Ottawa (Protocol OHRIe3562), McMaster University (Protocol #21-07-19), University of Western Ontario (Protocol #2022-023), University of Manitoba (Protocol #21-019), University of Alberta (Protocol #3381), and University of Calgary (Protocol AC23-0116). Elements of the PREPARE guidelines ^2^ and the Minimum Quality Threshold in Pre-Clinical Sepsis Studies (MQTiPSS) guidelines are considered in the design of this protocol. ^9^ The SPIRIT guidelines are followed, where applicable, in the reporting of this protocol, to optimally match the reporting practices use for human clinical trial protocols. ^10^ The SPIRIT checklist can be found on our Open Science Framework National Preclinical Sepsis Platform-01 (NPSP-01) project homepage, DOI 10.17605/OSF.IO/R5G7Y. ARRIVE guidelines will be used when reporting the final results of our study. Our study protocol has been registered in preclinicaltrials.eu (PCTE0000552). All protocol amendments will be reflected in updates to the registered protocol.

### Study design and setting

This is a randomized controlled multilaboratory preclinical study with three arms: sham + treatment (i.e., healthy animals), sepsis, and sepsis + treatment. The allocation ratio will be 1:2:2. The inclusion of a sham group will allow us to distinguish the effects of sepsis and its treatment from baseline physiological responses, providing essential control data for interpretation of study outcomes. Research staff will be blinded for disease induction, treatment administration, and outcome evaluation. All experiments will be carried out in animal facilities of six academic or research institutions across Canada. Table 1 lists the participating centers in the study.

**Table 1.** Participating centers and roles.

Center	Role
Ottawa Hospital Research Institute	Coordinating center; Conducting experiments
McMaster University	Conducting experiments
University of Western Ontario	Conducting experiments
University of Manitoba	Conducting experiments
University of Alberta	Conducting experiments
University of Calgary	Conducting experiments

### Eligibility criteria

*Inclusion criteria:* C57BL/6 mice at 10-12 weeks of age will be included in the study. Mice will be specifically bred for this study by the Charles River facility in Saint-Constant, Quebec, Canada where one male is paired with two females for breeding. Pups will remain with their parents until shipment to the designated centers.

*Exclusion criteria:* Prior to randomization, mice that do not pass routine health assessments by local veterinary staff will be excluded. Mice <16 g or >30 g will be excluded. No exclusions will occur after mice have been allocated to an experimental group.

### Experimental model: fecal-induced peritonitis (FIP)

The FIP model was selected as the first model to be tested by the NPSP. The FIP model only requires an intraperitoneal injection of fecal slurry for sepsis induction, which is less technically demanding than surgical models (e.g., cecal ligation and puncture). This results in less interoperator variability and can facilitate standardization of baseline conditions across multiple laboratories. To refine and optimize the model for this study, 24 pilot experiments were conducted, with some of the early results published by our group. ^11^ Although initial pilot studies employed a 72-hour experimental timepoint, an 8-hour endpoint model was believed to be more feasible for implementation by all participating laboratories given available personnel, intensity of monitoring, and funding, while still inducing a severity of illness reflective of acute sepsis. Additionally, all mice in the FIP pilot studies that received 0.75 mg/g of fecal slurry survived beyond 8-hours after sepsis induction, suggesting that the 8-hour endpoint model may ensure optimal survival and tissue collection in the mice.

*Preparation of fecal slurry:* To minimize batch to batch variability, the rat fecal slurry required for disease induction was prepared in bulk (100mg/mL in dextrose-glycerol) by one center (McMaster University), aliquoted, and stored at -80°C until use. The microbial composition of this batch of slurry was previously characterized. ^11^ Fecal slurry aliquots will be shipped on dry ice to all participating centers, where their frozen state will be verified upon receipt prior to immediate storage at -80°C. For sham animals, vehicle aliquots containing 5% dextrose (Fisher Chemical, Cat#: D16-3) in 10% glycerol (Fisher Bioreagents, Cat #: BP229-1) alone will be autoclaved and stored at -80°C in each center for use in the experiments.

*Mouse acquisition, housing and husbandry:* All mice for the NPSP-01 will be shipped to individual institutions directly from Charles-River at six weeks of age in 3-4 batches. Upon receipt, mice will be housed in groups of 2-3 per HEPA-filtered cages (segregated by sex). Housing will be equipped with corncob bedding (1/4-inch or 1/8-inch, irradiated or autoclaved), nesting material (Nestlet and crinkle paper), and one structure in the form of cardboard hut or dome. Each cage will be provided with chow (Teklad Irradiated Global 18% Protein Rodent Diet 2919) and water (autoclaved reverse osmosis). To minimize in-fighting/barbering, male mice in each cage will be from the same litter. Mice will be kept in the animal facility for a 4-6 week acclimation period prior to the start of the experiments.

### Interventions

All mice will receive a subcutaneous injection of buprenorphine (0.05 mg/kg body weight, Ceva, DIN: 02342510) for pain management at 4 hours post-induction, to meet current MQTiPSS recommendations ^2^ for animal welfare. Additionally, at 4 hours post-induction, mice in the treatment groups will receive imipenem-cilastatin (25 mg/kg body weight, Sandoz, DIN: 02358344) and Ringer's lactate (15 mL/kg body weight, Baxter, DIN: 00061085) in the same injection subcutaneously.

### Experimental groups

A total of 192 mice will be randomly allocated to one of the following three groups:

1. Sham + treatment (dextrose-glycerol injection at t = 0, treated with imipenem-cilastatin and Ringer’s Lactate at t = 4), both sexes; n = 36 mice.
2. Sepsis alone (fecal slurry injection at t = 0, no treatment), both sexes; n = 78 mice.
3. Sepsis + treatment (fecal slurry injection at t = 0, treated with imipenem-cilastatin and Ringer’s Lactate at t = 4), both sexes; n = 78 mice.

### Timeline

**Figure 1.**
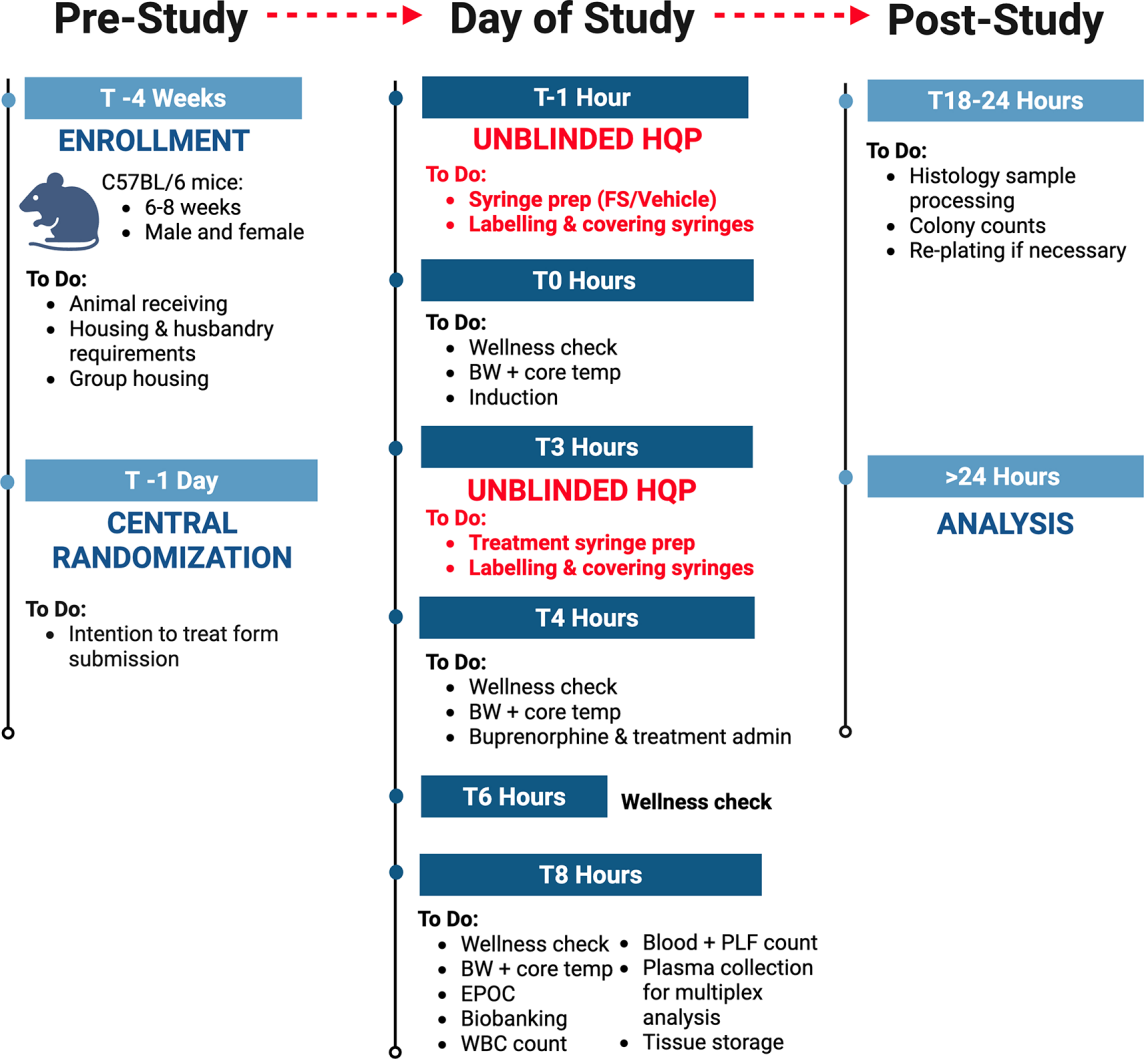
Experimental flow diagram for NPSP-C01 depicting pre-study, day of study, and post-study protocols for FIP sepsis induction in mice.

The schedule of enrollment, interventions, and assessments for NPSP-01, designed in accordance with SPIRIT guidelines, is outlined in Figure 1. Additional details on the study flow and the timeline of different procedures are illustrated in Table 2.

**Table 2.** Schedule of enrolment, induction, interventions, assessments, and biobanking for NPSP-01.

	Study period							
	Enrolment	Allocation	Post-Allocation					Close-out
Timepoint	-t _4 weeks_	-t _1 day_	t _0_	t _4_	t _6_	t _8_	>t _8_	t _4 months_
**Enrollment**								
Charles River shipment of mice to individual centers	X							
Receipt by animal facilities	X							
Housing and husbandry requirements	X							
Group caging (2-3 mice per cage)	X							
Central randomization		X						
Allocation		X						
Intention-to-treat form submission		X						
**Induction**								
Induction syringe administration			X					
**Interventions**								
Buprenorphine				X				
Imipenem-cilastatin and Ringer’s lactate				X				
**Assessments**								
Baseline variables (wellness check, body weight, and core temperature)			X					
Outcome variables (wellness check ± body weight and core temperature)				X	X	X		
Other data variables (blood chemistry analysis, white blood cell counts, blood and peritoneal lavage fluid bacterial counts)						X	Plasma multiplex analysis	
**Biobanking**								
Tissue and sample harvest						X	Processing & storage	X
Data transfer to online forms								X

### Randomization and blinding

Mice will be randomized in a 1:2:2 ratio to sham + treatment, sepsis alone, and sepsis + treatment arms. The allocation sequence will be computer-generated by a statistician at the central coordinating center using a stratified permuted block design with randomly varying lengths. Allocations will be stratified by laboratory and biological sex of animals to ensure balanced distribution across groups. Mice will be assigned a study identification number on arrival to the facility and the project coordinator will receive an email with the study ID number and treatment allocation. The randomization scheme will ensure allocation concealment from study personnel who are conducting the experiments in each laboratory.

Each center will designate one unblinded individual to be solely responsible for preparing induction and treatment syringes. This person will not participate in other study procedures. The project coordinator will send the allocation list to the designated unblinded individual at each center one day prior to the experiment. On the day of the experiment, unblinded individuals at each center will prepare the syringes and label with mouse numbers. The allocation list will be securely discarded post-syringe preparation. A separate, blinded individual will administer the syringes by matching to mouse numbers and thus remain blinded to the experimental groups.

*Assessments:* Except for the individual responsible for preparing the syringes at each center, all other personnel will remain blinded to group allocations. Consequently, all experimental protocols, animal monitoring, tissue collection, and point-of-care analyses will be conducted in a blinded manner. Data analysis will be performed without knowledge of center or group allocations, with unblinding occurring only after the final analysis is completed.

### Experimental procedures

Mice aged 10–12 weeks will be injected intraperitoneally with either the fecal slurry (0.75 mg/g body weight) or an equivalent volume of vehicle (dextrose-glycerol) under isoflurane anesthesia, targeting the right or left lower abdominal quadrant using a syringe with a 26-gauge needle. After injection, the abdomen will be massaged for 10 seconds, and the mice will be returned to their cages to recover. Heating blankets set at 37-38°C will be placed under half of each cage to support the mice in regulating their body temperature.

Overall health will be assessed with the murine sepsis score ^12^ modified for use in NPSP-01. Briefly, mice will be evaluated for locomotor activity, orbital tightening (indicative of pain or distress from clinical condition), fur appearance (reflective of grooming and hydration), and posture before handling for body weight or temperature measurements. This will be completed at baseline, 4, 6, and 8 hours post-induction. Each mouse will be evaluated at each timepoint by at least two blinded personnel and the calculated mean sepsis score will be used at each timepoint. Body weight and core temperature (as assessed by a rodent rectal probe thermometer) will be measured at baseline, 4 and 8 hours post-induction.

At 4 hours, mice will receive a subcutaneous injection of either buprenorphine alone or a combination of buprenorphine, imipenem-cilastatin, and Ringer’s lactate. At the study endpoint (8 hours post-induction) mice will be anesthetized with isoflurane and carotid blood will be collected into EDTA-coated tubes. A portion of the blood will be used for analysis of blood gas and chemistry, while the remaining blood will be used to measure white blood cell counts and bacterial load, and for plasma isolation. Animals will be euthanized under general anesthesia and then exsanguinated. Comprehensive biobanking will be performed with up to 20 samples collected per mouse (2-3 samples per tissue). These include plasma, peritoneal lavage fluid, tissues (brain, heart, lung, kidney, spleen, and muscle), and cecal content. Plasma will be prepared by centrifugation of blood at 5000 x g for 10 minutes and will be stored in aliquots of 20 to 100 μL at -80°C. Collected samples will either be stored at -80°C or fixed in formalin at 4°C for a minimum of 24 hours for future histology assessments. Formalin-fixed tissues will subsequently be washed in 1x PBS (Fisher BioReagents, Cat#: BP24384) and stored in either 0.1% sodium azide (VWR Avantor, Cat #: RC7144.8-16) with 30% sucrose (Sigma Aldrich, Cat#: S7903-1KG) (brain samples) or 70% ethanol (Greenfield Global, Cat#: P016EAAN) (all other tissues) for long-term storage.

### Protocol harmonization

To assess reproducibility and inter-laboratory variability in the NPSP-01 study, we will implement harmonized experimental conditions, including consistent mouse sourcing (as described above), animal care (e.g., cage conditions, husbandry, food and water), consensus based standard operating procedures, centralized biobanking protocols, and standardized supplies and devices across all participating labs. Multiple sources of heterogeneity and variation remain, to avoid the ‘standardization fallacy’, where excessive standardization can obscure real world variability and confound external validity. ^13^ The use of male and female mice, differences in facility environments as well as the number and experience of personnel in each lab will be uncontrolled variables. ^14^

*Training materials:* Each center will be responsible for ensuring that its research personnel are adequately trained in experimental techniques including injection protocols, animal handling, monitoring, and tissue collection. To facilitate this training, we have provided training videos and resources. Detailed standard operating procedures were created and shared with all members via a SharePoint (Microsoft, USA) repository to ensure consistent implementation across centers.

*Supplies and devices:* To reduce confounding due to equipment variability, all necessary supplies (e.g. tubes, vials, EDTA-coated tubes for blood collection, imipenem-cilastatin antibiotic (Sandoz Canada)) as well as devices including thermometers (INTELLIBIO, A-2205-00389), hemocytometers (Hausser Scientific Hemacytometer, #0267151B), Epoc analyzers (Siemens, Model PD470SH-B), and cartridges have been provided by the coordinating center to all participating centers.

### Outcomes

**Primary outcome**

Plasma IL6 will be assessed at the end of experiment (8 h) or at surrogate humane-endpoint of death, measured using a multiplex analysis (Eve Technologies, Calgary, Canada). IL6 is a well-established biomarker of systemic inflammation in sepsis and plays a critical role in the pathophysiology of the disease. Elevated plasma IL6 levels are strongly associated with disease severity, organ dysfunction, and mortality in both preclinical models and human sepsis patients; IL6 levels also have demonstrated sensitivity to therapeutic interventions. ^8, 15– 17^ Its consistent elevation in septic conditions and rapid decline with effective treatment make it a suitable primary outcome for evaluating inflammatory responses, as well as a useful biomarker to help guide translational efforts.

**Secondary outcomes**

*Clinical outcomes:* Murine sepsis score, body weight, and core temperature will be measured as described above. Mortality, evaluated by surrogate humane endpoints will also be assessed. Mice meeting any of the predefined humane endpoint criteria (body weight drop exceeding 20% from baseline, a core temperature drop exceeding 20% from baseline, inability to right when placed on their side, labored breathing, an average modified murine sepsis score greater than 2.5 for any two criteria, or a total average murine sepsis score greater than 9.4) will be humanely euthanized via isoflurane anesthesia with exsanguination, and their tissues will be collected for analysis. Those mice euthanized before study end will be included in an intention-to-treat analysis, using their most recent data for the analysis.

*Biomarker outcomes:* Bacterial load will be quantified in blood and peritoneal lavage fluid by plating samples on blood agar and assessing colony-forming units after incubation. White blood cell count will be measured manually by a hemocytometer as an indicator of immune response. Arterial blood gas and chemistry will be assessed with a point of care device (Epoc Blood Analysis System, Siemens Healthineers). Plasma inflammatory markers (granulocyte-macrophage colony-stimulating factor [GM-CSF], interferon [IFN] γ, IL1β, IL2, IL4, IL10, IL12p70, monocyte chemotactic protein [MCP]1, TNFα) and cardiovascular markers (pro-matrix metalloproteinase [MMP]9, plasminogen activator inhibitor [PAI]1, platelet endothelial cell adhesion molecule [PECAM]1, soluble platelet [sP]-Selectin, soluble endothelial [sE]-Selectin, soluble intercellular adhesion molecule [sICAM]1, thrombomodulin) will be measured by the multiplex analysis.

*Feasibility outcomes:* Several measures of feasibility, including study completion, technical success, biobanking completion, and protocol adherence will be assessed as follows:

Study completion: Defined as adherence to the allocation and successful plasma collection for IL6 analysis in ≥80% of mice.

Technical success: Defined by the occurrence of any of the following six technical issues in ≤20% of mice: Subcutaneous injection at T0, incomplete injection at T0, incomplete injection at T4, accidental organ punctures, arterial blood collection failures, and compromised blood quality (e.g., clotting). If multiple technical issues are observed in a single mouse, they will be recorded as a single event for the purposes of outcome assessment.

Biobanking completion: Defined as biobanking of ≥ 90% tissues in ≥ 80% of mice reaching the study endpoint (8 hours).

Protocol Adherence (Yes/No): Defined as meeting at least 90% of 15 specified criteria for each mouse in ≥80% of mice. Criteria include husbandry practices (such as the use of appropriate bedding, nestlets, crinkle paper, cardboard huts/domes, food, water, and ventilated cages), group caging, use of the correct fecal slurry batch, strict adherence to SOPs, maintenance of blinding procedures, appropriate time staggering, use of external heating, assessment of mice wellness by multiple individuals, and proper sample storage.

### Sample size

For this feasibility study, our sample size calculation is based on detecting differences in plasma IL-6 between septic and septic + treatment groups at each of the six participating centers (two-arm comparison with equal allocation) at the study endpoint. Sample sizes of 12 mice in each arm (total 24 mice per center) will achieve 80% power to detect a mean difference of 6,000 in fluorescence intensity using a two-sided t-test with equal variance at the 5% level of significance. We assumed a standard deviation of 5,000 based on pilot multiplex analysis data. An additional two animals per group will be included at each center to account for potential attrition. Thus, our total sample size allocated to the two treatment arms across 6 centers is 156 mice. An additional 36 mice will be allocated to the sham+treatment arm for a grand total of 192 mice across 6 centers.

### Statistical analysis plan

*Baseline description of the groups*

Descriptive analyses will be used to compare baseline characteristics of mice allocated to the different study arms. Measures of central tendency (e.g., mean or median) and dispersion (e.g., standard deviation or inter-quartile ranges) will be calculated for all continuous variables (e.g., body weight, core temperature, murine sepsis score) and counts and proportions for categorical variables (e.g., sex). As differences in treatment effects across centers is of specific interest, we will also explore differences in baseline characteristics of mice across centers using descriptive statistics.

*Analysis of differences between non-treated and treated septic mice*

Descriptive statistics will be used to describe outcomes in each arm (mean and standard deviation or median and inter-quartile range as appropriate) or frequency and proportion. Histograms and normal probability plots will be used to investigate skewness in continuous outcome variables and normalizing transformations will be applied as necessary. Additional descriptive analyses will characterize outcomes in each arm by center.

*Primary analysis*

Analysis of our primary outcome, plasma IL6, will be conducted using a linear regression analysis (after normalizing transformation if necessary). To obtain correct inferences, the model will include the stratification factors: sex and center as fixed covariates. Adjusted least square mean differences with 95% confidence intervals will be obtained from the model and used to compare mean plasma IL-6 levels at 8 h (or when humane endpoint is reached) between Sepsis and Sepsis + treatment groups (primary comparison).

*Inter-laboratory variability analysis*

To formally assess differences in treatment effects on mean plasma IL6 levels between laboratories, we will extend the primary analysis described above by including the interaction between center and treatment as a fixed effect into the statistical model. The statistical significance of the treatment by center interaction will be used to test the hypothesis that treatment effects differ by center. Least square mean differences from the model with 95% confidence intervals will be used to estimate the treatment effects at each center. We will use forest plots to visualize mean differences and 95% confidence intervals across the six laboratories and contrast them with the overall pooled finding to visually assess heterogeneity. Additional analyses will allow for extra variability in the pooled treatment effect estimates due to differences between centers by modeling center as a random effect and including center and center by treatment interaction as random terms in the model, provided the center by treatment variance component is positive. The random effects analyses will be used to quantify variability in outcomes between centers using intracluster correlation coefficients.

*Secondary analyses*

For all secondary outcomes, the effect of the treatment will be evaluated as for our primary analysis for continuous variables (i.e., mean differences and 95% confidence intervals). For dichotomous secondary variables (e.g., mortality), we will conduct logistic regression analyses adjusted for the stratification factors and obtain odds ratios and risk differences with 95% confidence intervals.

*Feasibility analysis*

Descriptive analyses will be carried out using classification in categorical variables and using means and SD in numerical variables.

*Additional subgroup analyses*

Subgroup analyses by male and female animals will be carried out for primary and secondary outcomes. These analyses will be carried out by adding interaction terms between sex and treatment to the statistical models and using least square mean differences from the model to estimate sex-specific treatment effects.

*Population analysis and missing data*

*Primary analyses:* Primary analyses will follow an intention-to-treat (ITT) approach, including all randomized animals to ensure a comprehensive evaluation of treatment effects. The presence of missing data will be limited through rigorous training but missingness can arise (e.g., in plasma IL-6 measurements due to insufficient blood collection). We will explore the potential impact of missing data on inferences by comparing characteristics of animals with missing outcome data and those with complete data. Multivariable logistic regression analyses will be used to explore factors associated with missingness under a Missing At Random assumption, i.e., that missingness depends only on observed characteristics. If any characteristics associated with missingness are identified, additional sensitivity analyses will be carried out by adjusting for these characteristics in the linear and logistic regression analyses.

We will also conduct a per-protocol analysis for animals that received injections at T0 intraperitoneally and all interventions (e.g. fluids and antibioitics) as detailed.

*Secondary analyses:* Several secondary analyses are being planned for NPSP-01, including assessment of laboratory personnel characteristics on the study outcomes, histological analyses of biobanked tissue, microbiome analysis of cecal contents, and assessment of the modified murine sepsis score. Prior to initiating these studies, protocols for each will be developed and posted.

### Study management

*Coordinating center*

The trial will be centrally coordinated by a project coordinator (FJ) based at the Ottawa Hospital Research Institute, under the supervision of the NPSP-01’s primary investigator (MML) and a senior scientist from the methods center (DAF). The study structure also includes principal investigators as well as personnel at each participating center. The project coordinator will maintain regular contact with participating laboratories prior to study initiation to ensure that all study infrastructure is in place and that personnel at each center have received adequate training. After each experimental day, the project coordinator will perform online monitoring of collected data to ensure validity and proper study execution, including intervention implementation, outcome measurement, biobanking, and sample storage. Statistical consulting, randomization, and analyses will also be conducted at the Methods Center of the Ottawa Hospital Research Institute.

### Data management

Study data will be collected and managed through a user-friendly interface built with React, with data stored in a PostgreSQL database hosted on Heroku. The backend, built with express, ensures reliable and secure data processing. Backup of data will be scheduled daily with Heroku PGBackups.

All data will be recorded on preformatted ‘mouse report forms’, which include designated fields for dates and IDs of responsible personnel. Each center’s principal investigator will review the forms for accuracy and completeness. Data forms will then be scanned and uploaded to center-specific folders in the study's SharePoint within 48 hours of collection. Data will also be uploaded to a web-based platform with automated completeness checks. A designated individual will transfer data to the online database, with a second individual verifying accuracy to minimize input errors. Once submitted, forms will lock to prevent any further modifications.

### Data monitoring

Quality assessments will be conducted by the coordinating center through remote monitoring. The project coordinator will verify the completeness of uploaded forms in SharePoint and randomly audit a selection of forms for consistency between the manual and electronic submissions for each center on the experimental dates. In the event of any inconsistencies, the project coordinator will inform the principal investigator of the specific center about discrepancies identified and work collaboratively to resolve issues.

### Access to data

During the course of the study, access to the data will be granted only for input and verification.

Upon completion of NPSP-01, a complete cleaned dataset will be prepared, together with its related dictionaries. This dataset will be made available on the publication of the scientific papers using an online data repository.

## Discussion

The NPSP-01 study will unite diverse expertise across various laboratories to address longstanding barriers in preclinical sepsis research and advance the field. Our evaluation of interlaboratory and intralaboratory variability will provide critical insights into variability thresholds, which are essential for designing future multilaboratory sepsis studies. By incorporating biological sex as a variable into experimental design, the NPSP-01 will also directly address the need for preclinical models that mirror heterogeneity of human sepsis. In addition, assessing how standard clinical interventions for sepsis (antibiotics and fluid resuscitation) affect this model will help establish a baseline to compare novel interventions in future studies. Furthermore, the rigorous implementation of harmonized protocols, randomization, blinding, and transparent reporting sets a high standard that supports the generation of high-quality and reliable data.

Beyond its immediate scope, NPSP-01 also establishes a scalable and versatile platform that can be readily adapted to test other models of sepsis. This platform could also be used to address pandemic response efforts and other emergent public health challenges requiring rapid and rigorous evaluation of therapies. By mitigating the risks associated with non-reproducible research, this platform has the potential to maximize the impact of scientific investments and streamline the translation of promising treatments to early-phase clinical trials.

## Disclosures

### Ethics approval and consent to participate

The study is approved by the University of Ottawa, Approval # OHRIe3562.

### Consent for publication

Not applicable.

## Data Availability

No data are associated with this article. SPIRIT Guideline checklists can be publicly accessed at National Preclinical Sepsis Platform-01 (NPSP-01) on Open Science Framework, DOI 10.17605/OSF.IO/R5G7Y. ^18^ Given the lack of reporting guidelines for animal protocols, we felt this was the closest relevant guideline.
