# Supplementary material for: Assessing the effects of fluids and antibiotics in an acute murine model of sepsis: study protocol for the National Preclinical Sepsis Platform-01 (NPSP-01) Study
Source: F1000Res. 2026 Apr 28;14:472. Originally published 2025 May 1. [Version 3] doi: 10.12688/f1000research.163800.3 (PMC13049416; doi:10.12688/f1000research.163800.3)
Supplement: Peer review history

# Reviewer report: Reviewer response for version 2

**DOI:** 10.5256/f1000research.196367.r458400

**Published:** 2026-04-03

**Authors:** Nelly Amenyogbe

**Affiliations:** 1 Dalhousie University, Dalhousie, Canada

The authors have improved the manuscript with a  thorough response to review comments. Below very minor points reg. grammar and procedural details.  The suggested changes refer to the reviewer questions as outlined in response to reviewer 1.

 Question 4. Would suggest to change “ *Thus, the 8 hour model also mitigates survivorship bias as killing all animals at this timepoint will help ensure data collection from a full cohort.”*

 -Instead of *“killing” please use “euthanizing”*

 Question 5. “Mice will be specifically bred for this study by the Charles River facility in Saint-Constant, Quebec, Canada *(Charles River barrier facilities K61 and K92 (specific pathogen–free). Animals will originate from breeding units (one male, two females) and wean according to standard procedures.”*

*-“weaned” instead of “wean”*

 Minor methods details:

 Question 7 (tail markings):  Are these tail markings via permanent tattoo?  Markers?  If so what kind?  For example there are specialized non-toxic markers made to identify mice (https://www.ketchum.ca/product.htm?pid=963227).  Would a product like this be utilized?

 Question 8.  Please elaborate on the standardized injection volume by body weight – e.g. are you using 10 microliter per g BW (so 250 uL for a 25g mouse?) Given that the IP injection is also extra fluids given to the animal, having this detail in the protocol will help others implement these details.

Is the study design appropriate for the research question?

Yes

Is the rationale for, and objectives of, the study clearly described?

Partly

Are sufficient details of the methods provided to allow replication by others?

Partly

Are the datasets clearly presented in a useable and accessible format?

Not applicable

Reviewer Expertise:

innate immunity, neonatal sepsis, vaccines

I confirm that I have read this submission and believe that I have an appropriate level of expertise to confirm that it is of an acceptable scientific standard.

# Response

**Published:** 2026-04-08

**Authors:** Manoj Lalu

**Affiliations:** Ottawa Health Research Institute, Canada

We thank the reviewer for their feedback. Below please find our responses to their comments:

**Question 4. Would suggest to change “ *Thus, the 8 hour model also mitigates survivorship bias as killing all animals at this timepoint will help ensure data collection from a full cohort.”***

**-Instead of *“killing” please use “euthanizing”***

**Response**: We thank the reviewer for this suggestion. However, following consultation with an animal welfare scientist, and in accordance with the CCAC Ethics Principles for Animal Use in Science ( https://ccac.ca/Documents/Standards/Guidelines/CCAC_Ethics_Principles_for_Animal_Use_in_Science.pdf) , we have retained the term “humane killing” in the protocol to clearly distinguish between the two procedures (see Glossary, p. 25). We have revised the protocol to ensure consistent use of this terminology throughout.

 Euthanasia − Killing an animal when the reason for terminating the animal’s life is for the benefit of the individual animal (this term is being phased out by the CCAC; humane killing will be used in all new and revised CCAC documents).

 Humane killing − The intentional ending of an animal’s life using methods that demonstrate respect for the animal and minimize negative welfare impacts.

**Question 5. “Mice will be specifically bred for this study by the Charles River facility in Saint-Constant, Quebec, **Canada *(Charles River barrier facilities K61 and K92 (specific pathogen–free). Animals will originate from breeding units (one male, two females) and wean according to standard procedures.”***

***-“weaned” instead of “wean”***

**Response**: Grammar is corrected now.

*“…will be weaned…”*

**Minor methods details:**

**Question 7 (tail markings):  Are these tail markings via permanent tattoo?  Markers?  If so what kind?  For example there are specialized non-toxic markers made to identify mice (https://www.ketchum.ca/product.htm?pid=963227).  Would a product like this be utilized?**

**Response**: Yes, we used the non-toxic markers for the tail marking (Fine Science Tools Inc.). This info has now been added to the paper.

*“…using tail markings (non-toxic markers (Fine Science Tools Inc.))…”*

**Question 8.  Please elaborate on the standardized injection volume by body weight – e.g. are you using 10 microliter per g BW (so 250 uL for a 25g mouse?) Given that the IP injection is also extra fluids given to the animal, having this detail in the protocol will help others implement these details.**

**Response**: We have added more details about the injection volume to the text.

*“Mice will receive approximately 15 uL of fecal slurry (50 mg/mL) per gram of body weight. Across the expected weight range of 16-30 g, the difference in administered fecal slurry volume will be up to 200 µL. As mice will be randomized across groups, this variation is expected to have minimal impact on experimental outcomes.”*

# Reviewer report: Reviewer response for version 1

**DOI:** 10.5256/f1000research.180201.r437806

**Published:** 2025-12-31

**Authors:** Arnaud Mansart

**Affiliations:** 1 Université Paris-Saclay, Montigny le bretonneux, France

Overall synthesis

 This study protocol describes a well-conceived and ambitious multicenter preclinical study that directly addresses several long-standing limitations in translational sepsis research. By adopting clinical-trial standards, the NPSP-01 study represents a meaningful methodological advance. Beyond its biological objectives, the study’s main value lies in establishing a scalable and reproducible framework for future preclinical trials.

 Major strengths

 A key strength of the protocol is its six-center randomized controlled design, which remains rare in animal research. This approach directly tackles issues of reproducibility and inter-laboratory variability, two major contributors to translational failure in sepsis. Modeling the study after multicenter clinical trials is therefore a notable and commendable advance.

 The methodological rigor is high. Centralized randomization stratified by center and sex, blinding at multiple stages, and clearly predefined primary and secondary outcomes all contribute to strong internal validity.

 The inclusion of clinically relevant standard-of-care interventions, particularly antibiotics and fluid resuscitation, substantially enhances construct and translational validity. Many experimental sepsis models omit these elements, limiting clinical relevance. Establishing a treated baseline is especially valuable for future evaluation of novel therapeutic strategies.

 The explicit inclusion of both male and female mice aligns well with current best practices and strengthens the biological relevance of the findings.

 Another major strength is the extensive feasibility and process evaluation. Metrics such as technical success rates, protocol adherence, and biobanking completion will generate highly informative data for the design of future multicenter preclinical studies, not only in sepsis but also in other disease areas.

 Finally, the authors strike a thoughtful balance between harmonization and real-world variability, avoiding excessive standardization. This reflects a sophisticated understanding of external validity and enhances the generalizability of the findings.

 Areas for improvement and clarification

 The relatively short experimental time frame, with an 8-hour endpoint, limits insight into the dynamic kinetics of sepsis and the progression of organ dysfunction. While pragmatic, this constraint should be clearly acknowledged when interpreting results and extrapolating to human sepsis.

 Important dimensions such as age-related dysfunction are not addressed. Although sex diversity is intentionally introduced, age (a major determinant of sepsis susceptibility, immune response, and outcome) is not considered.

 The protocol would also benefit from a clearer discussion of downstream decision rules. It is not specified what degree of inter-laboratory heterogeneity would be considered acceptable, or how such variability will inform future study design, sample size calculations, or protocol refinement.

 Additional methodological considerations

 Several sources of variability occurring before and after the experimental protocol deserve further attention. The sanitary status of the animal facilities prior to study initiation is critical, as undetected infections or contaminations will significantly influence outcomes. Environmental factors such as room temperature are not monitored, despite their impact on hypothermia and long-term survival in septic mice. These aspects could be documented.

 Internal variability within individual laboratories is not explicitly assessed. This could be evaluated using simple, pragmatic parameters all of which can serve as indicators of technical reproducibility.

 Finally, there is a potential procedural bias related to fluid administration. The sepsis group does not receive a dextrose–glycerol injection at T4, unlike the other groups, which may induce unnecessary differences in long-term hypovolemia. Does the buprenorphine injection adequately compensate for this difference?

 Conclusion

 Overall, this protocol represents a landmark step forward in preclinical sepsis research. Its greatest contribution may lie less in its biological findings and more in its rigorous, transparent, and reproducible trial-like framework. With additional analyses that explicitly account for, rather than avoid, sources of variability, the study has strong potential to shape the design of future multicenter preclinical investigations and improve translational success in sepsis research.

Is the study design appropriate for the research question?

Yes

Is the rationale for, and objectives of, the study clearly described?

Yes

Are sufficient details of the methods provided to allow replication by others?

Yes

Are the datasets clearly presented in a useable and accessible format?

Yes

Reviewer Expertise:

preclinical sepsis researches

I confirm that I have read this submission and believe that I have an appropriate level of expertise to confirm that it is of an acceptable scientific standard.

# Response

**Published:** 2026-02-06

**Authors:** Manoj Lalu

**Affiliations:** Ottawa Health Research Institute, Canada

**Arnaud Mansart**, Université Paris-Saclay, Montigny le bretonneux, France 

**Approved**

**info_outline**

**1. Overall synthesis**

**This study protocol describes a well-conceived and ambitious multicenter preclinical study that directly addresses several long-standing limitations in translational sepsis research. By adopting clinical-trial standards, the NPSP-01 study represents a meaningful methodological advance. Beyond its biological objectives, the study’s main value lies in establishing a scalable and reproducible framework for future preclinical trials.**

**Major strengths**

**A key strength of the protocol is its six-center randomized controlled design, which remains rare in animal research. This approach directly tackles issues of reproducibility and inter-laboratory variability, two major contributors to translational failure in sepsis. Modeling the study after multicenter clinical trials is therefore a notable and commendable advance.**

**The methodological rigor is high. Centralized randomization stratified by center and sex, blinding at multiple stages, and clearly predefined primary and secondary outcomes all contribute to strong internal validity.**

**The inclusion of clinically relevant standard-of-care interventions, particularly antibiotics and fluid resuscitation, substantially enhances construct and translational validity. Many experimental sepsis models omit these elements, limiting clinical relevance. Establishing a treated baseline is especially valuable for future evaluation of novel therapeutic strategies.**

**The explicit inclusion of both male and female mice aligns well with current best practices and strengthens the biological relevance of the findings.**

**Another major strength is the extensive feasibility and process evaluation. Metrics such as technical success rates, protocol adherence, and biobanking completion will generate highly informative data for the design of future multicenter preclinical studies, not only in sepsis but also in other disease areas.**

**Finally, the authors strike a thoughtful balance between harmonization and real-world variability, avoiding excessive standardization. This reflects a sophisticated understanding of external validity and enhances the generalizability of the findings.**

 We thank the reviewer for their careful review and insightful comments.  We have addressed their concerns below and in the manuscript. We believe the edits made should address outstanding concerns.

**2. Areas for improvement and clarification**

**The relatively short experimental time frame, with an 8-hour endpoint, limits insight into the dynamic kinetics of sepsis and the progression of organ dysfunction. While pragmatic, this constraint should be clearly acknowledged when interpreting results and extrapolating to human sepsis.**

**Response: **We thank the reviewer for this insightful comment. We acknowledge that the 8h experimental timeframe limits assessment of sepsis progression and organ dysfunction over longer periods. We have added this to the limitation section in the discussion.

 “ *There are several aspects that should be considered when applying the model described in this protocol. 1) The 8h experimental timeframe limits assessment of longer-term sepsis progression and organ dysfunction, and modifications would be required for studies addressing these aspects…*.”

**4. Important dimensions such as age-related dysfunction are not addressed. Although sex diversity is intentionally introduced, age (a major determinant of sepsis susceptibility, immune response, and outcome) is not considered.**

**Response:** We agree, however as first step we want to ensure feasibility in a younger, healthier cohort prior to considering more resource intensive experiments in comorbid and aged animals. We plan to extend the model in future studies to incorporate additional complexity. This has now been addressed as a limitation in the discussion.

 “… *2) The use of young animals does not capture age-related physiological changes or disease susceptibility; future adaptations incorporating aged animals, neonatal models, or relevant comorbidities may improve construct validity…”*

**5. The protocol would also benefit from a clearer discussion of downstream decision rules. It is not specified what degree of inter-laboratory heterogeneity would be considered acceptable, or how such variability will inform future study design, sample size calculations, or protocol refinement.**

**Response:** We agree that clearer downstream decision rules strengthen the protocol. There are no universally accepted thresholds defining “acceptable” versus “high” inter-laboratory variability in multicentre clinical trials (i.e., where these issues have been studied extensively). Rather, interpretation is context-dependent and informed by empirical distributions of site-level variance.

 We have now clarified how inter-laboratory heterogeneity will be interpreted using intraclass correlation coefficients (ICCs), and how these estimates will inform protocol refinement, sample size assumptions, and design choices for future multilaboratory studies.

 Statistical analysis plan:

 “The random effects analyses will be used to quantify variability in *the IL6* outcome between centers using intracluster correlation coefficients (ICCs). *Intracluster correlation coefficient estimates the proportion of total outcome variance attributable to between-laboratory differences. Consistent with practice in multicentre clinical trials, smaller ICC values will be interpreted as indicating limited between-laboratory heterogeneity, whereas larger values will prompt closer examination of protocol implementation and contextual sources of variability rather than being treated as a priori unacceptable*.”

 Discussion:

 “ *Estimates of interlaboratory variance from NPSP-01 may help inform the design of subsequent multilaboratory studies by guiding assumptions for sample size calculations, determining whether center should be modeled as a random effect, and identifying domains where additional harmonization or training may be required. Where substantial heterogeneity is observed, future protocols will need to consider incorporating targeted refinements, or modified designs to improve interpretability*.”

**6. Additional methodological considerations**

**Several sources of variability occurring before and after the experimental protocol deserve further attention. The sanitary status of the animal facilities prior to study initiation is critical, as undetected infections or contaminations will significantly influence outcomes. Environmental factors such as room temperature are not monitored, despite their impact on hypothermia and long-term survival in septic mice. These aspects could be documented.**

**Response:** We thank the reviewer for this comment. Procedures for temperature and humidity monitoring, as well as the cage change schedule, are defined in our protocols. This information has now been added to the manuscript:

 “ *Temperature and humidity will be documented within each animal facility. To maintain sanitary status, cage changes will be performed every 10-14 days, or earlier if cage soiling exceeds each centre’s standard thresholds, in line with local animal care procedures*.”

**7. Internal variability within individual laboratories is not explicitly assessed. This could be evaluated using simple, pragmatic parameters all of which can serve as indicators of technical reproducibility.**

**Response:** Intra-centre variability will be reported using 95% confidence intervals around the primary outcome (IL6) estimate. The approach was described in section “ *Inter-laboratory variability analysis” *in the manuscript.

**8. Finally, there is a potential procedural bias related to fluid administration. The sepsis group does not receive a dextrose–glycerol injection at T4, unlike the other groups, which may induce unnecessary differences in long-term hypovolemia. Does the buprenorphine injection adequately compensate for this difference?**

**Response:** None of the groups receive dextrose-glycerol at T4; only the treatment groups receive fluids and antibiotics, while the sepsis group does not. We acknowledge this as a potential limitation, but fluid resuscitation and antibiotics represent the primary intervention under study. Therefore, we could not compensate for administered volume in the sepsis group.

**8. Conclusion**

**Overall, this protocol represents a landmark step forward in preclinical sepsis research. Its greatest contribution may lie less in its biological findings and more in its rigorous, transparent, and reproducible trial-like framework. With additional analyses that explicitly account for, rather than avoid, sources of variability, the study has strong potential to shape the design of future multicenter preclinical investigations and improve translational success in sepsis research.**

 We again thank the reviewer for their positive comments and recognizing the value of our planned approach.

# Reviewer report: Reviewer response for version 1

**DOI:** 10.5256/f1000research.180201.r437807

**Published:** 2025-12-31

**Authors:** Nelly Amenyogbe

**Affiliations:** 1 Dalhousie University, Dalhousie, Canada

The manuscript describes a rigorous multi-centre preclinical protocol designed to assess the impact of iv fluids and broad-spectrum antibiotics on plasma IL-6 levels in an adult mouse model of polymicrobial sepsis.  The manuscript describes a thorough governance structure intended to meet the standards of clinical trials involving humans. This level of standardization both improves animal welfare and aims to improve the applicability of findings within preclinical models to human translation. Below are minor comments to improve the work.

**Introduction:**  The introduction can be written more clearly to describe the objectives of the work.  Currently, the introduction states that “For our initial study, NPSP-01, we are implementing procedural standardization and rigorous practices to establish baseline conditions for future investigations.” However, eventually in the methods section (where the challenge dose was justified) the authors reference their prior work (doi: 10.1186/s40635-023-00533-3) whereby multi-centre dose titrations were performed and the administration of fluids and antibiotics evaluated at 4 and 12 hours post sepsis challenge. In this manuscript, the early administration of Abics + fluids at 4h and at this dose rescued 100% of the mice. Those details are important to put the current work into context. For example, based on prior work, do the authors intend to later implement the sepsis model with 4h fluids and antibiotics with an 8 hour readout as a standard way to evaluate sepsis interventions in a clinical setting or is the current study simply evaluating the impact of the intervention on IL-6 levels?

**Exclusion criteria:**  Please elaborate on the welfare criteria put in place to exclude animals that arrive to the facility and indicate whether these are the same for all participating institutions.

**Endpoint selection**:  can the authors elaborate on the pilot experiments that informed the design of this pilot?  All mice survived to the eight hour timepoint.  However, When were the endpoints reached in these models?  How variable were the clinical scores of animals across different groups at this time?  What was the experiment LD for these pilot studies at the 0.75 mg/g dose?

**Mouse acquisition**:  At what age are the mice shipped to the respective institutions?  The authors wrote above that mice will remain with their dams (i.e. will not be weaned) until shipment to the laboratories.  Does the shipment time also represent the weaning time for these mice?

**Experimental groups**:  I would suggest to clarify here and in the flow chart sample size at each respective institution.  While eventually the authors clarify that the study size of 192 mice is the pooled sample size across all centres, it is not clear at this point nor is the sample size per site given in the Figure.

**Randomization and blinding:**  how are individual mice distinguished (e.g. ear notch? Another method?)

**Experimental procedures**:  What is the volume of injections given to each animal? Do the study centres also aim to synchronize the time of challenge? For euthanasia, the authors describe general anaesthesia and exsanguination. Can the authors comment specifically on the type, dose and duration of anaesthetic and whether this will also be constant across study centres?  Is there a secondary method of euthanasia following anaesthesia (e.g. cervical dislocation)?

**Technical success:**  Why would multiple events with the same mouse be counted as one event?  It would be more informative to analyze the frequency of each specific event separately across the study.

**Figure**:  A flow chart that illustrates the sample numbers by treatment group for each study centre would be more informative, and can still contain the same information as was already placed within the overview figure.

**Discussion:**  The discussion is very brief.  The authors can describe some of the potential challenges to deploying a standardized early treatment model to evaluate sepsis interventions.  For example, if the plasma IL-6 levels in the fluid and antibiotic treated groups is dramatically reduced, then would this readout allow for the detection of benefit from other interventions?  How would this reflect the timing of IV fluid and antibiotic support used in the human setting, if known?  Given that the team had previously identified that IV fluids and antibiotics given 12 hours post challenge are not effective, interventions that can effectively treat or prevent sepsis in cases where IV fluids or broad spectrum antibiotics are not given early enough in the challenge course would need to be evaluated in a different context.

 Can the authors comment on whether plasma IL-6 as an indicator of sepsis severity varies across age groups? For example, in neonates the specificity of IL-6 for sepsis detection is generally lower than for adults (see doi: 10.1007/s00431-025-06409-w). The authors could suggest future work to evaluate the model in other physiological states (e.g. during pregnancy, ageing, neonates).

**Other comments:**

 Can the authors comment on why rat cecal slury was selected instead of mouse cecal slurry?  How might the species difference impact the overall validity of the model?

 How was the 4-hour timepoint for treatment administration selected?

Is the study design appropriate for the research question?

Yes

Is the rationale for, and objectives of, the study clearly described?

Partly

Are sufficient details of the methods provided to allow replication by others?

Partly

Are the datasets clearly presented in a useable and accessible format?

Not applicable

Reviewer Expertise:

innate immunity, neonatal sepsis, vaccines

I confirm that I have read this submission and believe that I have an appropriate level of expertise to confirm that it is of an acceptable scientific standard, however I have significant reservations, as outlined above.

# Response

**Published:** 2026-02-06

**Authors:** Manoj Lalu

**Affiliations:** Ottawa Health Research Institute, Canada

**Nelly Amenyogbe**, Dalhousie University, Dalhousie, Canada 

**Approved With Reservations**

**info_outline**

**1. The manuscript describes a rigorous multi-centre preclinical protocol designed to assess the impact of iv fluids and broad-spectrum antibiotics on plasma IL-6 levels in an adult mouse model of polymicrobial sepsis.  The manuscript describes a thorough governance structure intended to meet the standards of clinical trials involving humans. This level of standardization both improves animal welfare and aims to improve the applicability of findings within preclinical models to human translation. Below are minor comments to improve the work.**

**Response:** We thank the reviewer for their careful review of our protocol and providing thoughtful comments. We have addressed concerns below and in the manuscript. We believe the manuscript has been significantly strengthened as a result.

**2. Introduction:  The introduction can be written more clearly to describe the objectives of the work.  Currently, the introduction states that “For our initial study, NPSP-01, we are implementing procedural standardization and rigorous practices to establish baseline conditions for future investigations.” However, eventually in the methods section (where the challenge dose was justified) the authors reference their prior work (doi: 10.1186/s40635-023-00533-3) whereby multi-centre dose titrations were performed and the administration of fluids and antibiotics evaluated at 4 and 12 hours post sepsis challenge. In this manuscript, the early administration of Abics + fluids at 4h and at this dose rescued 100% of the mice. Those details are important to put the current work into context. For example, based on prior work, do the authors intend to later implement the sepsis model with 4h fluids and antibiotics with an 8 hour readout as a standard way to evaluate sepsis interventions in a clinical setting or is the current study simply evaluating the impact of the intervention on IL-6 levels?**

**Response:** Our previously published dose-titration study was not conducted as a fully implemented multicenter investigation, but rather served as an initial evaluation of fluid and antibiotic administration at 4 and 12 hours following sepsis induction to inform the development of NPSP-01. During subsequent feasibility assessments, we identified logistical and technical constraints that limited consistent implementation of this 72h model. To ensure standardization and feasibility across sites, the consortium therefore adopted an 8-hour experimental model for our multicenter investigations. We have now added this to the main manuscript for more clarity:

*“As an initial step, we published a 72 hour dose-titration study evaluating fluid and antibiotic administration at 4 and 12 hours following sepsis induction to inform the development of NPSP-01* *^8^* *. During subsequent feasibility assessments, we identified logistical and technical constraints that limited consistent implementation of this model across laboratories. To ensure standardization and feasibility, we have adopted an 8 hour experimental model for our multicenter investigations. At this time point mice develop a septic phenotype, however, this precedes the 60-90% mortality we observed in our initial study. Thus, the 8 hour model also mitigates survivorship bias and helps ensure data collection from a full cohort.”*

**3. Exclusion criteria:  Please elaborate on the welfare criteria put in place to exclude animals that arrive to the facility and indicate whether these are the same for all participating institutions.**

**Response:** We do not have *a priori* criteria for this purpose, and institutes will determine general mice wellness based on their institutional animal care standards and veterinarian recommendations. This has been now added to the manuscript:

*“* *At six weeks of age, trained Charles-River personnel will perform dual verification prior to packaging and transport to participating centers. Upon receipt, each institute will assess general mouse wellness based on its routine animal care standards and veterinary recommendations.”*

**4. Endpoint selection:  can the authors elaborate on the pilot experiments that informed the design of this pilot?  All mice survived to the eight hour timepoint.  However, When were the endpoints reached in these models?  How variable were the clinical scores of animals across different groups at this time?  What was the experiment LD for these pilot studies at the 0.75 mg/g dose**?

**Response:** Fecal slurry (from 2 batches prepared in one center) at 0.75 mg/g resulted in heterogeneous mortality between the two centers. Mortality ranged from 10–60% at 16 h post-FIP, increased to 30–90% at 24 h, and reached 60–90% by 72 h post-FIP. Corresponding changes in MSS over the 72-h period for the 0.75 mg/g dose at each center are shown below.

 Img1: *https://f1000research-files.f1000.com/linked/781635.163800_Img1.jpeg*

 (From: Sharma et al. Intensive Care Medicine Experimental (2023) 11:45; https://doi.org/10.1186/s40635-023-00533-3)

 This has now been added to the introduction for context. 

 “ *At this time point mice develop a septic phenotype, however, this precedes the 60-90% mortality we observed in our initial study. Thus, the 8 hour model also mitigates survivorship bias as killing all animals at this timepoint will help ensure data collection from a full cohort.”*

**5. Mouse acquisition:  At what age are the mice shipped to the respective institutions?  The authors wrote above that mice will remain with their dams (i.e. will not be weaned) until shipment to the laboratories.  Does the shipment time also represent the weaning time for these mice?**

**Response:** Mice will be weaned at 3-4 weeks of age and shipped to the study centers at 6 weeks of age. These details have now been clarified:

 “Mice will be specifically bred for this study by the Charles River facility in Saint-Constant, Quebec, Canada *(Charles River barrier facilities K61 and K92 (specific pathogen–free). Animals will originate from breeding units (one male, two females) and wean according to standard procedures.”*

 “ *…At six weeks of age, trained Charles-River personnel will perform dual verification prior to packaging and transport to participating centers.”*

*“… Mice will be kept in the animal facility for a 4-6 week acclimation period prior to the start of the experiments to minimize stress and cage aggression. Experiments will begin when animals are 10-12 weeks old*.”

**6. Experimental groups:  I would suggest to clarify here and in the flow chart sample size at each respective institution.  While eventually the authors clarify that the study size of 192 mice is the pooled sample size across all centres, it is not clear at this point nor is the sample size per site given in the Figure.**

**Response:** Thank you for the suggestion. The sample size (n) per center will be between 28 and 36. The exact numbers will be determined by feasibility and human resources at each center. This is similar to clinical trials, where a goal sample per centre is declared, but final numbers are determined by feasibility and resources.  Figure 2 has been revised to reflect this information.

 Img2: https://f1000research-files.f1000.com/linked/781637.163800_Img2.jpg

 Revised Figure 2.

**7. Randomization and blinding:  how are individual mice distinguished (e.g. ear notch? Another method?)**

**Response:** Tail marking was used to distinguish individual mice. This has now been clarified in the manuscript.

*“Mice will be assigned to each center randomly by Charles River on the day of shipment. Mouse ID will be assigned using tail markings on the day of the experiment. Identification will be independently verified by an additional HQP before the start of the experiment, during outcome assessments, and biobanking to ensure accuracy.”*

**8. Experimental procedures:  What is the volume of injections given to each animal? Do the study centres also aim to synchronize the time of challenge? For euthanasia, the authors describe general anaesthesia and exsanguination. Can the authors comment specifically on the type, dose and duration of anaesthetic and whether this will also be constant across study centres?  Is there a secondary method of euthanasia following anaesthesia (e.g. cervical dislocation)?**

**Response: **The following information has been added to the manuscript:

*“All centres will initiate experiments in the morning and stagger induction time so that animals reach their endpoints within a narrow time window (8 ± 10 minutes) to minimise potential timing-related confounders.”*

 “ *Injection volumes will be adjusted according to body weight and experimental group.”*

*“Euthanasia procedures are standardised across centres. Terminal anaesthesia will be induced with isoflurane (5% in an induction chamber followed by 1–2% via nose cone in oxygen at 0.5–1 L/min). The duration of anaesthesia will be recorded in standardised *‘ *mouse report forms*’ *. Cervical dislocation will be used as a secondary method of euthanasia following anaesthesia*.”

**9. Technical success:  Why would multiple events with the same mouse be counted as one event?  It would be more informative to analyze the frequency of each specific event separately across the study.**

**Response:** Feasibility was assessed at the level of the experimental unit (mouse) and was a process-level question. Multiple deviations could occur across different, unrelated SOP categories, but any single event in a mouse was sufficient to classify it as a deviation. This is sensitive – a single deviation would count as a process failure.

**10. Figure:  A flow chart that illustrates the sample numbers by treatment group for each study centre would be more informative, and can still contain the same information as was already placed within the overview figure.**

**Response:** Thanks for the suggestion. As noted above, the total number of animals per center will range between 28 and 36, with exact numbers determined by feasibility and capacity at each center. Consequently, the exact number of mice per treatment group at each center cannot be specified at this stage. These details will be provided once the study is completed.

**11. Discussion:  The discussion is very brief.  The authors can describe some of the potential challenges to deploying a standardized early treatment model to evaluate sepsis interventions.  For example, if the plasma IL-6 levels in the fluid and antibiotic treated groups is dramatically reduced, then would this readout allow for the detection of benefit from other interventions?  How would this reflect the timing of IV fluid and antibiotic support used in the human setting, if known?  Given that the team had previously identified that IV fluids and antibiotics given 12 hours post challenge are not effective, interventions that can effectively treat or prevent sepsis in cases where IV fluids or broad spectrum antibiotics are not given early enough in the challenge course would need to be evaluated in a different context.**

**Response:** We thank the reviewer for these important comments. As this manuscript describes the protocol, we have intentionally kept the discussion brief. In order to avoid any bias toward the study outcomes, we plan to address potential challenges, translational considerations, and limitations in detail in the main paper once the data are collected, analyzed, and interpreted.

 Nonetheless, based on this comment and a similar suggestion by the second reviewer, we have now added a paragraph on the limitations.

 “There are several aspects that should be considered when applying the model described in this protocol. 1) The 8h experimental timeframe limits assessment of longer-term sepsis progression and organ dysfunction, and modifications would be required for studies addressing these aspects; 2) The use of young animals does not capture age-related physiological changes or disease susceptibility; future adaptations incorporating aged animals, neonatal models, or relevant comorbidities may improve construct validity;  3) additional readouts may be needed when evaluating other therapeutics, as outcomes depend on the characteristics of the animal model and the effects of standard of care treatments, such as fluids and antibiotics.”

**12. Can the authors comment on whether plasma IL-6 as an indicator of sepsis severity varies across age groups? For example, in neonates the specificity of IL-6 for sepsis detection is generally lower than for adults (see doi: 10.1007/s00431-025-06409-w). The authors could suggest future work to evaluate the model in other physiological states (e.g. during pregnancy, ageing, neonates).**

**Response:** We thank the reviewer for this suggestion. This aspect has now been added to the limitation paragraph in the protocol. Please see the response to Question 11.

**Other comments:**

**13. Can the authors comment on why rat cecal slury was selected instead of mouse cecal slurry?  How might the species difference impact the overall validity of the model?**

**Response:** Rat cecal slurry was selected by our group to provide a single, standardized batch that could be used across all centres. Rats were also selected to minimize animal use - if we were to use mouse cecal slurry a very large number of mice would need to be euthanized to produce an adequate volume. We acknowledge that species differences between the slurry donor (rat) and experimental subjects (mouse) could potentially affect some aspects of the model. We have now added this information to the manuscript:

 “ *Rats were selected to minimise animal use, as producing an adequate volume of fecal slurry from mice would require euthanizing a substantially larger number of animals*.”

**14. How was the 4-hour timepoint for treatment administration selected?**

**Response:** The 4h timepoint for treatment administration was selected based on pilot data and the aims of the study. Administering at 4 hours post FIP allows interventions to be given neither too early avoiding masking the disease phenotype, nor too late when their efficacy would be minimal. This timing also reduces handling stress by aligning treatment with the scheduled 4h body weight and core temperature assessments and a single analgesic dose sufficient for pain control for the rest of the experiment, thereby avoiding additional injections in the 8h model and supporting animal welfare.
